# Some Further Observations on Why Minor Gynecological Operations Fail of Anticipated Results1Read before the Section on Obstetrics and Gynecology, Buffalo Academy of Medicine, October 23, 1906.

**Published:** 1906-12

**Authors:** Sigmund Goldberg

**Affiliations:** Buffalo, N. Y.; Gynecologist to the German Hospital and Consulting Gynecologist to the German Hospital Dispensary. 508 East Eagle Street


### Some Further Observations on Why Minor Gynecological Operations Fail of Anticipated Results.[1]

By SIGMUND GOLDBERG, M.D., Buffalo, N.Y.
Gynecologist to the German Hospital and Consulting Gynecologist to the German Hospital Dispensary.

I READ a paper before this section of the Academy, March 24, 1903, entitled, "Why Minor Gynecological Operations Fail to give Anticipated Results." I stated as a general proposition that a minor gynecological operation was one which did not involve the opening of the peritoneal cavity. I also stated in a general way that, given an injury or specific infection of the female genital tract not being immediately repaired and specific infection arrested, it would produce an endometritis which would not limit itself after one or two years to any one portion of the endometrium, but would spread through continuity of tissues, thus involving the adnexa.

I further claimed at the time that the repair of the cervix or treatment of the other minor gynecological conditions would fail to produce perfect cure, the disease after this length of time having gone beyond local treatment, surgical or otherwise. I gave as my conclusions at that time:

1. That all so-called minor gynecological operations to be of any permanent benefit must be performed within at least one year from time of the infliction of the original lesion.

2. That after the passage of about a year either Alexander's operation, trachelorrhaphy, or currettage, or all combined, will be insufficient to restore a patient's perfect health.

3. The reason for such failure in the great majority of such cases, after one year, is because the adnexa are sure to be involved.

4. The best course will be to do some operative procedure intra-abdominally.

I do not consider the above conclusions a plea for the radical opening of the abdomen irrespective of time or condition, but rather for the performance of minor gynecological work much earlier than is the custom of many men at the present time, or not at all except as correlative to more radical work. It is not my intention to bring before you a complete disquisition of this subject as I did on the occasion referred to, but I bring it before you in this manner because of the general interest involved in these subjects, and for the purpose of creating a discussion which may prove both interesting and beneficial.

---

1. Read before the Section on Obstetrics and Gynecology, Buffalo Academy of Medicine, October 23, 1906.

I quoted Hofmeier as having conducted studies on fresh uteri removed from mammals in which the conditions ought to be the some as in the human female, but he also examined organs removed at the operating table and at once immersed in a warm saline solution. In several of these latter he demonstrated conclusively, by removing strips of endometrium and placing them under the microscope, that minute particles of charcoal were invariably carried by the ciliary movement from the fundus toward the os.

This observation of Hofmeier seems at least to be in harmony with an intelligent design of nature, by which obstacles are interposed to the easy invasion of the upper reaches of the genital tract. But, under the stress of injury or infection, these frail barriers are no longer effective or at least, in a great measure, have lost their potency and this once lost, progressive endometrial infection traveling on and infecting the adnexa, is the next step in the progress of the majority of the sexual ills of womankind. Next we find, due to the constant irritation and consequent increased weight of the uterus, a complication of adnexial disease, a retroversion or flexion or both; and this condition I consider the most common complication of adnexial disease.

Therefore, this condition generally receives the most attention, because of its alleged influence on the general health; but to my mind, these various retropositions of the uterus are, per se, insufficient to intelligently account for all the symptoms usually ascribed to them. True, they may give some feeling of discomfort, some difficulty of locomotion, and in many cases not even this. The more active symptoms generally ascribed to retroposition, such as pain, metorrhagia, sacralgia, and more or less permanent invalidism, are due to the adnexial involvements. It seems to me the height of folly to repair the cervix and fasten the uterus by the Alexandrian method or any of its modofications. For, after one year, we may feel morally certain that the seeds of disease are sown in the tubes and ovaries, although we may not always be able to positively demonstrate the exact condition by the usual method of examination.

J. Sprecht says that the early stage of pyosalpinx or salpingitis is hard to recognise because it follows immediately upon, or is associated in conjunction with, endometritis, the endometritis masking the symptoms of tubal disease. It is on account of our diagnostic fallibility that so many minor gynecological operations, such as the Alexander, currettage and trachelorrhaphy, result with only a modicum of benefit, and the cause, therefore, being the failure to recognise the early stages of disease in the adnexa.

In substantiation of the above, I have culled from current literature, and shall bring before you some of the thoughts of a few of the most prominent gynecologists, showing the change of opinion concerning this matter, which has taken place in very recent years, and especially with regard to the importance of a retroverted or a retroflex uterus as a producer of symptoms of pelvic disease.

In the New York Medical Journal of April 14, 1906, J. M. Baldy, of Philadelphia, in an article entitled, "Intraperitoneal Shortening of the Round Ligaments," says: "I may consider myself fortunate this evening in being limited to the discussion of the intraperitoneal shortening of the round ligaments for backward displacement of the uterus, because I have long since reached the conclusion that other surgical procedures are inadequate, and their further discussion a waste of time."

When in addition to this consideration is given the fact that so many of the complications are of such a nature as to render the certainty of a prior diagnosis doubtful, a consideration of the operation at all is useless, especially as there are so many other procedures which accomplish without exception everything which the Alexander operation does, and more. The modification of the Alexander operation, which is best and is a vast improvement on the parent procedure, is the Goldspohn method. The fact, however, that this procedure involves two incisions, each at a point at which greater damage may be done than at the site of a single median incision, is quite sufficient to dispose of it, unless there is nothing else to take its place.

Ventrofixation and ventrosuspension are far superior to the Alexander procedure, as they recognise the unassailable position that the operation is performed in view of the complications, allow of a correct diagnosis, and permit an intelligent treatment of the real cause of the symptoms. The displacement itself falls into insignificance, excepting that its existence presupposes a displacement of the ovaries; the ovaries in time become cirrhotic, and this condition eventually is the most potent factor in the production of symptoms for which the patient applies for relief.

Inflammation, adhesions, and other complications form a large group in which a cure of the inflammation, or adhesion, or whatever other complications obtain, will end the case whether or not the uterus be displaced. The mere displacement of the uterus will not continue the symptoms. They allow of a treatment of the complications which in most instances is the treatment of the displacement; Alexander's does not. I have seen too many patients in whom perfect mechanical results were obtained, and still the symptoms persisted, either because the ovaries were hopelessly involved, or a mistake in judgment had been made in operating in a non-surgical case, often in a neurasthenic.

Further, in a discussion of this question before the Obstetrical Society of Philadelphia, March 1, 1906, Dr. George M. Boyd, said, "If it were possible to diagnosticate a case of uncomplicated retrodisplacement, then the Alexander operation has a field of usefulness which hardly any one can question."

Dr. John C. DaCosta did not think that any operation for old chronic retrodisplacement of the uterus was a satisfactory one, unless the abdomen was opened, for otherwise it could not be known what changes had taken place. In the Alexander operation, the risk was taken of doing a great deal of damage.

Dr. Daniel Longaker said that the treatment of retrodisplacements of the uterus consisted in the treatment of their complications. The results in the days of the Smith-Hodge pessary were probably quite as satisfactory as those of the Alexander operations employed today. One of the gentlemen, Dr. Noble, believed the diagnosis of uncomplicated retroversion could be made.

Dr. Fischer said he desired to congratulate Dr. Noble upon his diagnostic ability. He remarked that he had served an apprenticeship in gynecology extending over a good many years, during which period he had come into contact with men of large experience in gynecological work, and he was forced to say that there had been comparatively few cases of retrodisplacement with symptoms in which conditions of some kind were not found that were unsuspected previous to opening the abdomen. From all accounts, he was almost forced to admit that there were men who could palpate a kink in a *normal* ureter and outline other equally attenuated structures in the pelvis but he, likewise, knew that every now and then a man of this class made a mistake in diagnosis where he happened to succeed. On the other hand, he at times found that he was in error while other men's opinions were confirmed, and it was not an infrequent occurrence to find abdominal section reveal abnormalities that were separately diagnosticated by different men, while each in turn might have failed to recognise that which was diagnosticated by any one of his confreres.

If, therefore, Dr. Noble's diagnostic ability was of the character intimated, he believed the time had come for many to take a course in gynecology under his instructions. If, as Dr. Baldy had said, it was only the complications which gave rise to symptoms, he would inquire the excuse for one man's doing over 200 Alexander operations, for it is well known that women rarely consulted doctors concerning pelvic condition without first experiencing pelvic symptoms. In reference to the Alexander operation, he thought it scarcely suitable except for one of Dr. Noble's "extraordinary capabilities in the diagnosis of intrapelvic conditions."

And again, in an article published in the Medical Record of March 24, 1906, entitled, "The Uterus and Ovary of Neurasthenia," Robert L. Dickinson, of New York, says in reference to retroversion that there is one retroversion to every five cases in this series. This is about the usual average among gynecological patients. Among parous women, one in ten exhibited marked retroversion. Of these ten, all but one have been anatomical cures, yet failures as far as striking improvement to the general condition goes. Pessary and operation have permanently replaced all but one uterus, and to the pelvic distresses there has been afforded more or less local relief, as of dysmenorrhea and menorrhagia, but the other symptoms persist. The menstrual upset occurs still. The patient comes back asking for pelvic treatment. "It is to be remembered always," to quote his prize words, "that it is not, primarily, the amount of displacement, or flexion, that counts; it is the amount of inflammation, hemorrhage, or enlargement produced." These are the criteria as to the stress to be laid on the mechanical defect.

And still again, Frank C. Hammond, of Philadelphia, in an article published in the New York Medical Journal, August 18, 1906, entitled "A Report of a Year's Work in the Gynecological Service of the Samaritan Hospital, Including the Technique," makes this significant statement: "In the treatment of backward displacement of the uterus, we never employ Alexander operations, always depending upon one of the intraperitoneal procedures.

The foregoing indicates the trend of modern gynecological thought concerning this question, as found in the public medical press, and in the discussions of our most prominent medical men. There is also a change in our most modern gynecological textbooks, but before demonstrating that to you, let me read an extract on retroversion from an article in the American Textbook of Gynecology, giving the following symptomatology: Women with retroversion or retroflexion, complain more of backache and a dragging sensation in the pelvis than of any other symptoms. These may be so great as to amount to actual inability to walk. Leucorrhea is a prominent symptom, the discharge being milky or purulent. As a result, erosions of the cervix may occur. In septic or inflamed uteri, every movement is felt in the tender organ. Defecation is difficult and often painful, hence postponed as long as possible. Costiveness results with the common accompanying train of anorexia, foul breath, and the like. Dragging upon the bladder often causes the sphincter vesicæ to leak, and dribbling of urine occurs upon laughing or exertion. Pains down the front of the thighs are frequent, and are increased on motion. Occipital headache and burning pain in the nuchæ, inability to concentrate the thoughts, melancholia, hysteria, and peevishness are common reflex nervous phenomena. The endometrium commonly becomes hypertrophic, and gives rise to increased menstrual flow. This, however, is not painful as a rule, owing to the fluid condition of the blood and patency of the canal."

In Skene's treatise on diseases of women, a similar symptomatology is given. It is refreshing to turn from this conglomeration of hodge-podge to the saner writings of some of the modern authors, as shown in Montgomery's textbook on gynecology. Under the head of symptoms, we find: Retroversion causes few symptoms. The discomfort in the majority of cases arises from complications. Patients may have marked retroversion without experiencing any inconvenience or being aware of the condition until it is brought to their knowledge. Inflammatory complications produce a sensation of weight or dragging, as if everything were about to protrude when the patient stands or walks. The menstrual flow is increased, producing menorrhagia; occasionally, there is an irregular bloody discharge, or the intermenstrual intervals are shortened or, as a result of the coexisting catarrh, the patient will have a profuse leucorrhea.

In the Reference Handbook of the Medical Sciences, Vol. VIII, there is found the following:—Expert knowledge and skill are required to recognise and properly appreciate many of the conditions referred to, while even under an anesthetic, adhesions of the pelvic viscera, if in the form of long bands, will often escape detection. Inasmuch as the uterus has a tendency, when its position is altered by a force from without, to return to its original position, the elastic pressure exerted upon the pessary used, will be likely to produce injurious results. And then, on the other hand, if the normal position be maintained, pressure and traction upon the diseased tissues nearby, will ultimately increase the distress of the patient. An uncomplicated backward displacement of the uterus, for example, is a comparatively rare condition, and, like other displacements, were it not accompanied by increase in the size of the organ, endometritis, salpingitis, chronic peritonitis, pelvic congestion, varicose veins of the broad ligament, and imperfect circulation in lymph channels, it would produce comparatively little disability. While, therefore a displacement of the uterus may be the most easily demonstrated feature in a given case, it cannot be too strongly urged, that attention must first be given to the correction of complicating conditions, and especially to the removal of all adhesions, before the replacement of the uterus, by any form of support, is to be undertaken.

In view of all the above, I feel more than ever justified in asserting that the so-called minor gynecological operations fail of anticipated results, because the time for their performance has long passed, and I plead for an examination of all patients at least one year following parturition or after some specific infection. After about one year, it will be too late for any permannent benefits to be realised, and I agree with the authorities quoted, that only a laparotomy with ocular demonstration and radical treatment, can promise a complete and permanent cure.

508 East Eagle Street.

